# Perceptions of child death in Jigawa State, Nigeria: a mixed-methods study on how sociocultural nuances shape paediatric mortality reporting

**DOI:** 10.1080/16549716.2022.2120251

**Published:** 2022-11-03

**Authors:** Kristen Nicole Andersen, Funmilayo Shittu, Abdullahi Magama, Tahlil Ahmed, Damola Bakare, Julius Salako, Rochelle Ann Burgess, Carina King

**Affiliations:** a Department of Global Public Health, Karolinska Institutet, Stockholm, Sweden; b Department of Pediatrics, University of Ibadan, Ibadan, Nigeria and University College Hospital, Ibadan, Nigeria; c Save the Children International, Jigawa, Nigeria; d Save the Children UK, London, UK; e Institute for Global Health, University College London, London, UK

**Keywords:** Qualitative, verbal autopsy, vital statistics, religion, Nigeria

## Abstract

**Background:**

Vital statistics are critical for effective public health and monitoring progress towards child survival. Nigeria has the highest global under-five mortality rate; however, deaths are often under- or misreported.

**Objective:**

We explored perceptions of child deaths and socio-cultural factors influencing the reporting of child deaths in Jigawa State, Nigeria.

**Methods:**

We conducted a triangulation mixed-methods study in Kiyawa local government area, Jigawa, including: four focus group discussions (FGDs) with 8–12 women, six key informant interviews (KII) with Imams, and process data from 42 verbal autopsies (VAs) conducted with caregivers of deceased children. Data was collected between November 2019-April 2021. Purposive sampling was used to recruit FDG and KII participants and two-stage systematic and simple random sampling was employed to recruit VA participants. Qualitative data was analysed using content analysis; VA data was described with proportions.

**Results:**

Five categories emerged from FGDs: culturally grounded perceptions of child death, etiquette in mourning and offering condolence, formal procedures surrounding child death, the improving relationship between hospital and community, and reporting practices. Women expressed that talking or crying about a death was not culturally accepted, and that prayer is the most acceptable form of coping and offering condolence. Many women expressed that death was God’s will. These findings correlated with VAs, in which visible signs of emotional distress were recorded in 31% of the interviews. Three categories emerged from KIIs: religion as part of formal procedures surrounding child death, communities support the bereaved, and multilayered reasons for unreported deaths. Imams serve a key role as community leaders, involved in both the logistical and religious aspects of their community, though they are not involved in mortality reporting.

**Conclusion:**

Religion plays a central role in burial practices, community mourning rituals, and expression of grief, but does not extend to reporting of child deaths. Imams could provide an opportunity for improving vital registration.

## Background

Accurate and complete vital statistics data, including birth and death registration, are critical for the development, deployment, and evaluation of public health policies [1]. Additionally, such statistics are needed to track progress towards targets and indicators laid out in the United Nation’s Sustainable Development Goals (SDGs), particularly those related to child health [1]. An estimated 7.5 million children died in 2019, many of preventable or treatable causes [2]. However, this figure is reliant on modelled estimates compiled from diverse data sources, which may reflect an underestimation of the true scale of child mortality, as many low-income settings lack functioning civil registration systems [1–3].

Child death is a profoundly sensitive and emotional event that can give rise to interpersonal tensions, such as blame surrounding abortion, miscarriage, and stillbirth [4,5]. Grief experiences following child loss are largely shaped by cultural and religious beliefs, as well as social norms that dictate acceptable bereavement practices [5]. How death is perceived and understood can in turn impact the language that is used to describe child loss and behaviours surrounding mortality reporting [6–9]. For example, stillbirths have been shown to be grossly underreported in low-income settings and are unaccounted for in most global mortality reports, including the burden of disease and SDGs [4]. This data gap has significant ramifications for the allocation of resources towards prevention policies and public health interventions [2,4].

Nigeria has the highest recorded under-5 mortality rate (117 per 1,000 births) globally. This includes both neonatal mortality (36 per 1,000 births) and infant mortality (74 per 1,000 births) [10]. Many structural factors including financial and infrastructure constraints have contributed to chronic underreporting of vital events in Nigeria [11]. Because of the perceived benefits of birth and marriage registration, these events are easier to register and have been more systematically registered in Nigeria [12]. Death, in contrast, has been shown to be the most under-reported category of vital statistics, with only 10% of the deaths formally reported in 2017 [12]. Moreover, even when deaths are reported, the cause of death is often not included, as many deaths occur outside of medical facilities [3,13]. Verbal autopsies can be used to increase accurate mortality statistics and assign a cause of death when medical certification is unable to be performed [12].

Given Nigeria is a socio-culturally diverse setting, sub-national data on experiences with child loss and child mortality reporting could refine locally adapted systems. A previous study in Nigeria reported a cultural script of silence surrounding child death whereby women were encouraged to downplay the loss of a foetus or infant to prevent another such tragedy from occurring [5]. Similar findings have been reported from Tanzania, with a study reporting that women concealed pregnancy, particularly in early stages, as child death heightened women’s vulnerability to social and physical harm; the social risk to report impacted survey data [14]. However, given the high mortality observed in Jigawa State, we did not find evidence specifically for this setting. Therefore, we aimed to explore perceptions of child death and sociocultural factors that may influence the reporting of child death in Jigawa State, Nigeria, in order to inform data collection processes and tools for child mortality surveillance.

## Methods

We conducted a concurrent triangulation mixed-methods study (QUAL + quan), informed by Obst et al.’s (2020) socio-ecological model of grief [15]. Data sources included process data from verbal autopsies (VA, n = 42; personal level), community focus group discussions with women (n = 4; interpersonal level), and key informant interviews with Imams (n = 6; community level). This study was conducted as part of the formative research phase of the INSPIRING Jigawa randomised controlled trial (ISRCTN: 39,213,655), between November 2019 and April 2021 [16].

### Study setting

This study was conducted in two administrative wards (one peri-urban and one rural) within the Kiyawa Local Government Area (LGA), Jigawa state. The predominant ethnic groups in this region include Hausa and Fulani and the population is mostly Muslim and governed by Sharia law [17]. Jigawa State is among the poorest states in Nigeria with an estimated 87% of the population living below the national poverty level in 2019 [18,19]. Educational attainment and literacy rates in the region are also low. In 2018, an estimated 75% of women and 37% of men aged 15–49 had completed no education, and 18% of women and 58% of men in the region were literate, defined by the ability to read all or part of a sentence. The under-five mortality rate in Jigawa State was reported to be 213 per 1,000 live births in 2018 [19].

### Sampling strategy

A purposive sampling design was used to recruit study participants for both the FGDs and key informant interviews. To include as many diverse perspectives as possible, two different groups were targeted: those living in peri-urban settings (Kiyawa ward) and those in rural settings (Maje ward). Recruitment was pragmatic and not based on reaching saturation. FGD participants were recruited from women who had previously participated in community conversation (Kiyawa) and concept testing (Maje), which focused on the INSPIRING project’s intervention design and acceptability through a participatory intervention adaption process; the topic of child mortality perceptions and reporting were not a focus of prior group discussions. Attendance lists from previous group discussions were used to contact participants with the help of gatekeepers from both wards. Due to the esteemed position of Imams in the community, researchers selected and contacted Imams through the district heads of each ward.

A two-stage random sampling strategy was employed to identify households with an eligible child's death for verbal autopsy. First, systematic random sampling was used to recruit compounds from all villages in Kiyawa LGA, taking an EPI approach, with sampling proportionate to village size. Within the recruited compounds, simple random sampling was used to select one woman with a child under-five to interview. Any woman from this sample who had reported a death of a child under-five years, including stillbirths, in the prior 12 months was eligible for a VA (Appendix A). This timeframe was chosen to minimise recall bias but ensure a culturally appropriate period had passed since the death. Families were approached by the study data collectors at their homes to take part.

### Data collection

#### Focus group discussions

Two rounds of two focus group discussions (FGDs) were conducted in each ward (4 FGDs in total), with 8–10 women in each group. The same group of 8–10 women were included in round one and two FGDs. Topic guides for these discussions were created in collaboration between local research assistants and experienced qualitative researchers (FS, RAB and CK). All FGDs were conducted in Hausa, audio recorded, transcribed, and translated into English by female Nigerian research assistants, who all have previous experience with qualitative data collection. The primary data collector (FS) was responsible for training research assistants and supervising data collection. These discussions focused on questions related to pregnancy, birth, and the process around child death and mourning; how death is talked about and perceived in the community, what happens after someone dies, and explanations of death in this context (Appendix B and C). In order to mitigate the time-burden for participants, data collection was conducted in convenient but private locations to ensure confidentiality. Participants were given reimbursement for transportation expenses and a small token (e.g. drink and biscuits).

#### Key informant interviews

Key informant interviews were conducted with major Imams within Maje (n = 3) and Kiyawa (n = 3) wards, those involved in leading Friday prayers. Imams were selected because of their trusted role within the community and their ability to provide further context on the role of religion in the community and in relation to child death. Many were engaged in disseminating health information to the community and to other Imams presiding in small neighbouring mosques. The topic guide for these interviews was written by KNA, in collaboration with FS, CK and RAB. As with the FGDs, all interviews were conducted in Hausa, transcribed, and translated into English by Nigerian female research assistants, who had previous experience with qualitative data collection. Data collection was conducted in convenient but private locations to ensure confidentiality and participants were reimbursed for transport costs and offered a simple token of appreciation (drinks and biscuits). Interviews focused on questions related to Imams’ role within the community, and specifically their role in child death (Appendix D).

#### Verbal autopsy

VAs were conducted using the World Health Organisation 2016 VA instrument, with four sections: open narrative, closed questions, care-seeking, and VA process questions [20]. The process data included demographic information about the VA respondents (who was interviewed, main respondent vs. other respondents), information about the child who died (age and sex of the child, location of death), and visible signs of emotional distress (e.g. crying), as determined by the interviewer. A cause of death was recorded if the VA respondent explicitly stated what they thought their child had died from, including non-medical causes; we did not conduct any further analysis of the VA data to assign a probable cause of death. VAs were conducted by female data collectors with a clinical background with previous experience conducting interviews within these communities. Interviews were done within the home and sought to speak to the primary caregiver of the child.

### Data analysis and synthesis

#### Qualitative

We analysed the FGDs and key informant interviews separately, using content analysis for both datasets [21]. Analysis of these sources was performed separately because of the differences in questions asked in order to independently assess individual, interpersonal and community factors related to child death and mortality reporting. Content analysis was performed inductively whereby raw text was reviewed for emerging patterns that were subsequently coded into sub-categories and categories (an example is given in Table 1). We focused on the manifest content (low abstraction, low interpretation) given the research aim and richness of the data. The unit of analysis for all qualitative material was paragraphs of text spoken by participants. Several rounds of coding were completed by KNA, and the codes, sub-categories, and categories were discussed and agreed upon between KNA and CK, and shared with FS and RAB for review. Coding for all qualitative data was performed using NVivo version 12 [22].

**Table 1.** Example of the qualitative content analysis process.

Participant	Quote	Code	Sub-category	Category
Participant 1 (FGD 1, Kiyawa)	*“Honestly in this town we see death as an act God, once it is your turn you will be taken whether you like it or not, whether he is in authority or not.’*	Death as inevitable or as an act of God	Non-medical perceptions of cause of death	Culturally grounded perceptions of child death
Participant 7, FGD 1 Kiyawa	*‘The truth is, if it is not prayer that one wants to offer on behalf of the dead or what God instructs about the dead then, too much talking is not good’*	Excessive talking about death is taboo	Prayer is the best way to show support and offer condolence	Etiquette in mourning and offering condolence
Participant 5, FGD 2 Maje	*‘The reason why we report or share details of the death of a child is because people will know what exactly brought about the death to avoid spreading wrong information and also get help in order to avoid the spread of the disease’*	Mortality reporting as preventative public health measure	Motivation for reporting to a hospital	Child mortality reporting practices
KII 1, Maje	*‘The difference is that a child has no sin on him so his prayer is just a minor one but that of an adult who has a lot of sin on him we will pray for Allah to forgive all his wrong doings’*	Appropriate prayer depends on age	Funeral rites for children are less elaborate than for adults	Religion as part of formal procedures surrounding child deaths

#### Quantitative

Description of the VA process data was conducted, using counts and percentages. All analyses were performed using STATA, version 16 [23]. Qualitative and quantitative data were analysed separately, and findings were triangulated in the discussion section.

## Results

### Verbal autopsies

A total of 48 eligible deaths were identified, and 42 VAs were successfully completed. Reasons for exclusion include: participant relocation (n = 2) and death occurred outside of the study timeframe (n = 4). Demographic and VA process information is reported in Table 2. Neonatal deaths and stillbirths accounted for 19% (n = 8) of the deaths, while older children aged 12–59 months accounted for 45% (n = 19) of deaths. Visible signs of emotional distress were reported in 31% (n = 13/42) of the VAs; of these, four interviews had to be paused.

**Table 2.** Demographic and verbal autopsy information (n = 42).

Demographics, deceased child		N (%)
Sex	Female	17 (40)
	Male	25 (60)
Age at death*	<1 month^†^	8 (19)
	1–12 months	14 (33)
	12–59 months	19 (45)
Location of death	Hospital	8 (19)
	Home	33 (79)
	En Route	1 (2)
Cause of death, reported	Anaemia	1 (2)
by caregiver	Asphyxia	1 (2)
	Fever/ Febrile Convulsion	3 (7)
	Teething	1 (2)
	Jaundice	2 (5)
	Malaria, breathlessness	1 (2)
	Malnutrition	1 (2)
	Not reported	32 (76)
Verbal autopsy data N (%)		
Main respondent	Mother	36 (85)
	Father	5 (12)
	Maternal grandmother	1 (2)
Other respondents	Partner (mother/father)	47 (38)
(n = 125)	Grandparent	17 (14)
	Other female relative	13 (10)
	Other male relative	30 (24)
	Neighbour	16 (13)
	Other	2 (1)
Care-seeking	Yes	25 (60)
	No	17 (40)
Location first sought care	Private pharmacy	2 (8)
(n = 25)	Govt. primary care	14 (56)
	Private hospital	1 (4)
	Govt. hospital	4 (16)
	Traditional care	4 (16)
Health facility > 2 hours	Yes	1 (2)
From home	No	38 (90)
	Don’t Know	3 (7)
Visible signs of emotional	Yes, crying	0 (0)
Distress	Yes, long silence	11 (26)
	Yes, other signs	2 (5)
	No	29 (69)
Interview paused due to	Yes, once	4 (31)
Emotion^‡^	Yes, more than once	0 (0)
(n = 13)	No	9 (69)

* In one record, the date of death was recorded incorrectly, and age of death was not determined

† This category includes stillbirths

‡ This question was only asked to those who displayed visible signs of emotional distress

### Focus group discussions

FGDs involved 20 women total and lasted from 45 to 90 minutes. Five categories emerged from these discussions, with considerable overlap of ideas between the two geographical wards (Table 3).

**Table 3.** Schematic presentation of categories and sub-categories from focus group discussions with community women.

Category	Sub-category
Culturally grounded perceptions of child death	Child death triggers an acute emotional response Language used for death Non-medical perception of causes of death Young children are grieved equally
Etiquette in mourning & offering condolence	Prayer is the best way to show support and offer condolence Role of the community in mourning Young children who die are mourned differently Only positive attributes about the deceased can be expressed Taboos
Formal procedures surrounding child death	Religious rituals Community participation
Improving relationship between hospital and community	Formal discussions about child death Greater trust in hospital for pregnancy and birth
Child mortality reporting practices	Location of child death influences reporting Hospital role in child death and reporting Motivation for reporting to a hospital

#### Category 1: culturally grounded perceptions of child death

Four sub-categories emerged within perceptions of child death, including: emotional responses to child death, language or synonyms used for death, non-medical perceptions of cause of death, and perceptions of young children who die. Child death was universally associated with pain, sadness, grief, or pity. Several respondents also spoke about how death triggers memories of the deceased and reflections on one’s own mortality. The local terms commonly used to describe death were ‘loss’, ‘transfer’, or ‘separation’. Participants often expressed a curiosity or speculation as to the cause of death. Non-medical explanations for cause of death were widespread and included: cold weather, rainy season, and humidity contributing to fever, convulsion, and ultimately death. For example:

This convulsion that occurs during the wet season starts with making the child’s body heavy and they will be showing signs of being frightened, and then in less than two minutes the child will faint and in most cases before getting to the hospital the child is dead (Participant 8, FGD 1 Kiyawa)

Fever and rainy season are mostly the causes that lead to the death of children (Participant 6, FGD 1 Maje)

Several participants expressed the view that death is inevitable or an act of God, which was often expressed as saying a person died because their ‘time was up’. The grief and sadness of losing of a child was universally perceived to be equal to that of an adult, but child death was perceived differently as being unexpected:

The death of a child is abnormal but when an adult dies it is normal because he/she has reached the number of their days here on Earth (Participant 5, FGD 2 Maje)

#### Category 2: etiquette in mourning and offering condolence

In discussions about offering condolence, five sub-categories were coded: the role of prayer, community participation in mourning, differing perceptions of mourning for children, the need to speak positively about the deceased, and taboos. Offering prayer was globally viewed as an expectation and cultural norm when showing sympathy to the family of the deceased. Several participants described that prayer was prioritised over other expressions of grief, including talking and crying, for example:

Avoid commotions and all sort of lamentations, instead one should just pray and recite words from the Quran and pray that the deceased finds eternal rest with Allah” (Participant 5, FGD 1 Maje)

The truth is, if it is not prayer that one wants to offer on behalf of the dead or what God instructs about the dead then, too much talking is not good (Participant 7, FGD 1 Kiyawa)

The community was described as playing a supportive role throughout the mourning process. When a child dies, the death is announced in the mosque or door to door, so that community members have the opportunity to visit with relatives of the deceased and offer condolences. One participant from Maje ward spoke about how neighbours and well wishers would bring food as an offering. Additionally, participants described how taking part in burial or mourning rituals was sometimes motivated by the desire to receive a reward from God. Thus, anyone in the community is welcome to offer condolences and take part in burial rituals:

Anyone that wants to be blessed even if you are not invited and you have the opportunity to go (Participant 7, FGD 1 Maje)

The mourning process was perceived differently for young children, with children under three years old mourned for a shorter duration than adults, if at all. Additionally, behaviour during life impacted how the deceased was spoken about after death, and several participants reflected that only positive words were allowed to be spoken about the dead. This was linked to discussions about taboos in offering condolences, and that it was taboo to gossip about the dead, ask about how they died, or speak ill of them in any way:

It is forbidden to gossip about someone that has died (Participant 5, FGD 1 Kiyawa)

It is not acceptable to ask what killed the child (Participant 6, FGD 1 Maje)

#### Category 3: formal procedures surrounding child death

Two sub-categories emerged from discussions on formal burial practices surrounding child death: religious rituals and community participation in burial. Religious practices include washing and preparing the corpse for burial as described by this quote,

In the beginning when a child dies, the child will be given the holy bath, then washed and covered in the burial cloth (Likafani) and/or praying mat before taking the child to the graveyard for burial while others bury at home (Participant 7, FDG 1 Kiyawa)

There was some contradiction in the responses about who was responsible for washing the corpse. While it was clear that religious knowledge was necessary in order to perform this task, several participants said that close family members or other community members could prepare the body for burial:

It depends, some will say that the woman that received the birth of the child should come and wash the child, while some people will say a relative will or the senior wife of the house. In some cases someone else or the imam should wash the child (Participant 9, FGD 1 Kiyawa).

#### Category 4: improving relationship between the hospital and community

In discussions about the interactions between the hospital and the wider community, two sub-categories emerged: formal discussions about child health and increasing levels of trust for hospitals managing pregnancy and birth. Women in both Kiyawa and Maje noted that formal discussions about child health and illness do occur and often involve hospital committees, community leaders, and government authorities. These discussions often centre around understanding causes of death or securing essential medications and seemed to be a changing norm:

Before we thought [death was] caused by the witches and wizard but now, because of the awareness we have, we sit-down and discuss, and see the cause of the child death (Participant 3, FGD 1 Maje)

The discussions concerning the deaths of children are what have made the government provide drugs that helps in protecting the children from diseases that usually kills them (Participant 7, FGD 1 Kiyawa)

Participants also noted a greater trust in the hospital system when dealing with pregnancy and birth. This was attributed to greater levels of education and community awareness of the importance of antenatal care and of delivering in the hospital, in case of potential complications. One participant from Kiyawa explained,

Before in this town no matter the hours it will take a woman to labour she will not be taken to the hospital, some have even lost their lives due to that, but now it is not like that (Participant 6, FGD 1 Kiyawa)

#### Category 5: child mortality reporting practices

Three sub-categories emerged from the discussions on child mortality reporting practices: influence of location of death on reporting, the role of hospitals in child death and reporting, and motivations for reporting to a hospital. Participants from both Kiyawa and Maje wards reported that if a child dies at home, the death is not reported. Deaths are only reported when the hospital is involved. Women in Kiyawa agreed that the role of hospitals in child death is primarily to confirm death or determine the cause of death, particularly in the case of unusual circumstances such as accidents or sudden deaths. One participant from Kiyawa shared,

Some people do report to the hospital because they doubt the reality of the child’s death. It’s not until they take the child to the hospital to confirm whether the child has died or not, before they will believe that the child has died (Participant 3, FGD 2 Kiyawa)

If a child dies as a result of fainting, strange illnesses and similar things are the ones that we do report to the hospital in order to find ways to avert future occurrences died (Participant 3, FGD 2 Kiyawa)

Reporting was often explained as secondary to receiving information and support from the hospital. Only one participant explained the motivation for reporting child death from a community protection perspective,

The reason why we report or share details of the death of a child is because people will know what exactly brought about the death to avoid spreading wrong information and also get help in order to avoid the spread of the disease (Participant 5, FGD 2 Maje)

### Key informant interviews

Six key informant interviews were conducted with Imams in Kiyawa (n = 3) and Maje (n = 3) wards of Kiyawa LGA. All Imams were male. The discussions lasted from 30 to 45 minutes and three universal themes emerged from the interviews. Categories and sub-categories are presented in Table 4.

**Table 4.** Schematic presentation of categories and sub-categories from key informant interviews with Imams.

Category	Sub-category
Religion as part of formal procedures surrounding child death	Imam role in funeral rites Funeral rites for children are less elaborate than for adults
Communities support the bereaved	Community engagement in funeral rites Shared prayer
Multilayered reasons for unreported deaths	Current practices for mortality reporting External responsibility for mortality reporting Barriers to mortality reporting

#### Category 1: religion as part of formal procedures surrounding child deaths

In discussions on formal burial practices for child deaths, two sub-categories emerged: Imam’s roles in funeral procedures and differing funeral practices for children. Imams in both Kiyawa and Maje wards described their role in funerals as orchestrating both the religious and logistical aspects of burial. Additionally, they are sometimes called upon to confirm death. An Imam from Maje described,

My role is to show people how the burial is done as we learned from our holy books because there are certain ways the Prophet thought us on how to go about burying the dead, and at a funeral if I see them doing anything contrary to the teaching of the hadith, I try to correct them (KII 1, Maje)

Imams also placed great importance on their role in leading prayer and providing religious teachings at funeral ceremonies. One Imam from Maje spoke about his role in educating others to pay condolence visits and offer prayers to the deceased. However, an Imam from Kiyawa also spoke of other motivations for prayer and against the tradition of paying religious scholars to pray for the dead over a certain duration of time. He explained,

Prayers for a dead person have no limit. It is not something you do for somedays and then you stop. The dead person needs to be remembered in prayers always. I always try to explain to people that a dead person needs to be prayed for always without any limitation. I feel gathering scholars for this prayer is a form of show off for people to see that you have prayed for your deceased (KII 1, Kiyawa)

Traditional burial practices for children were described as being less elaborate than those of an adult. In particular, Imams from Kiyawa explained that infants and younger children who die are often permitted to be bathed and prepared religiously by their mothers before being buried within the family compound. Moreover, how well a person is known often determines the size of a funeral gathering; thus, funerals for children are often smaller and less elaborate. Many Imams also emphasised how their own role shifts when a child dies, and they offer different funeral teachings and prayers. This difference was explained by an Imam from Maje who said,

The difference is that a child has no sin on him so his prayer is just a minor one but that of an adult who has a lot of sin on him we will pray for Allah to forgive all his wrong doings (KII 1, Maje)

#### Category 2: communities support the bereaved

In discussions about the role of the community in supporting those in mourning, two sub-categories emerged: community engagement in funeral rites and shared prayer. When a child dies, relatives announce the death to the community. One Imam noted that if the relatives of the deceased are poor, community members will supply the family with the traditional covering for the corpse. Imams from both Kiyawa and Maje emphasised the community’s role in congregating for shared prayer after a death,

People gather after the death has been announced in the community. We proceed to making arrangements for the burial, we pray for the person (salah) and we use such avenue to create awareness to people in the community, since it’s a gathering of different people in the community (KII 3, Kiyawa)

#### Category 3: multilayered reasons for unreported deaths

Three sub-categories emerged from discussions on the reporting of child deaths. These included current mortality reporting practices, external responsibilities, and barriers to reporting deaths. In both Kiyawa and Maje wards, Imams noted that the mosque is not involved in recording deaths in the community. In Kiyawa, Imams shared that deaths are reported but that the responsibility is external and often determined by governmental or nongovernmental organisations that partner with and train community leaders rather than religious leaders. In Maje ward, Imams shared that, to their knowledge, deaths are not recorded at all. Several reasons were provided for the lack of mortality reporting including illiteracy, the lack of a religious need to record deaths, and the absence of district-level mandates. Another reason that emerged was the absence of a historical precedent, particularly within the mosque. One Imam shared,

We have never seen or heard our fathers or forefathers recording deaths before. I have never even heard that people or mosques record deaths before now (KII 2, Maje)

## Discussion

In this mixed-methods analysis, we explored perceptions of child death to examine how the broader socio-cultural environment impacts experiences of child loss and the practice of mortality reporting in Jigawa State, Nigeria. In our analysis, there were several points of convergence across data sources that emerged as the main results for this study. The first point of convergence was around the outward displays of emotion, with most verbal autopsy respondents (69%) displaying no visible signs of emotional distress during the interview. This finding was linked to responses from FGDs during which women expressed that although losing a child generated a sense of deep sadness or pain, talking about the dead and the emotions such loss provoked was not culturally common. Literature from Ghana and Nigeria similarly reported a culture of silence surrounding infant loss, whereby social and cultural norms dictated that the best way to cope was to minimise thinking about child death and to forget the loss as soon as possible; women are often discouraged from talking about or seeing the corpse of their dead foetus [24,25]. Such norms have been shown to be motivated by a fear of exacerbating psychological harm for mothers and impairing their future fertility prospects [24,25].

The second point of convergence was around defining causes of death, with women in FGDs describing death as secondary to God’s will. This was most often expressed by saying that a death occurred because a person’s ‘time was up’. Similar fatalistic beliefs have also been observed in Ghana [24]. Research in Nigeria has shown that religious acceptance of loss is common and can act as a double-edged sword by both serving as a comforting internal coping mechanism and a shield preventing women from fully understanding the circumstances leading to the death of their child or from being able to question the loss [5]. This could explain why the cause of death was only reported by a quarter of caregivers in the Vas, and that among the causes given some suggest misconceptions in medical causes (e.g. teething).

Caregiver response and behaviour following child death is clearly influenced by strong cultural norms in this context, including the potential for gendered dynamics to influence how death is discussed. The most frequent secondary respondents in verbal autopsies were the main respondent’s partner or other male relatives, and it is important to consider that gendered power imbalances may be present when multiple respondents are interviewed together, affecting how emotions are displayed. Previous literature has shown that child death can generate interpersonal tension that can threaten marriages [5,14]. Moreover, women can be vulnerable to blame and stigma following child death [5,14]. Although respondents were able to choose who was present at verbal autopsy, women may not have the agency to exclude their husband or other household members from the process. This is supported by research in Ghana that highlighted confidentiality among household members as a central concern among VA informants [26]. Dynamics between interviewer-interviewee are also important to consider, as some evidence suggests that respondents may not feel comfortable sharing their emotion with outsiders [14,27]. While shame or blame related to child death were not raised in this study, further research is needed to investigate respondent vulnerability and gendered power dynamics in the context of verbal autopsy.

Differing perceptions and cultural norms regarding young children were observed throughout this study. For the Vas, only 19% of deaths were attributed to neonates and stillbirths. This is quite a low proportion when compared to statistics from the 2018 Demographic and Health Survey, which reported 22% of deaths in children under-five are among neonates – and exclude stillbirths [19]. This suggests respondents under-reported deaths amongst younger children. Previous studies have shown underreporting of neonatal deaths and stillbirths is widespread in settings with poor civil registration systems; secrecy surrounding such losses is often driven by the desire to avoid social stigma and blame [14,28]. However, we were unable to confirm if these were the underlying reasons for under-reporting in this context from our qualitative data.

Differing practices for younger children were also observed in both FGDs and key informant interviews. Women expressed that burial and mourning practices are age based, and young children are mourned for less duration or not at all. Additionally, Imams described that young children are permitted to be religiously bathed and buried by family members within their own compound, a practice, which may facilitate the invisibility of these children in mortality reporting [28]. This has important implications for vital statistics reporting as these deaths may be easily missed by current systems and could be an opportunity for Imams to collaborate with existing surveillance structures.

Despite being deeply integrated into the community and taking responsibility for both practical and religious aspects of funerals, Imams reported not being involved in mortality reporting. The reason given for this was not because of religious restrictions, but that it simply has not been done before. We found 79% of deaths in this study occurred at home (Table 2), similar to prior VA findings in Nigeria (72% neonates, 72% 1–59 month olds) [29]; approaches, which engage Imams could therefore improve reliable mortality reporting outside of facilities. The women stated that some deaths were reported to health facilities, but these may not be representative and could bias formal vital statistics. Community-based reporting initiatives have improved mortality reporting in several settings, including Mali and Malawi [30–32], and the importance of religious leaders supporting mortality reporting has been highlighted elsewhere [33,34]. The CHAMPS network provides examples of incorporating religious leaders into mortality surveillance [33,35]; however, it is important to explore the acceptability of this role among Imams in this Nigerian setting. Further, CRVS strengthening measures would still need to focus on strategies to improve underreporting of deaths among neonates and stillbirths, as Imams may not be aware of these deaths.

There are three key limitations to this study: first, recruitment and sampling for qualitative data was pragmatically driven and not based on reaching saturation and men were not included in FGDs. We are aware that gender is important in cultural constructs of mourning and grief, which limited the analysis and discussion on gender in this context. Secondly, we were unable to conduct interviews with community leaders who were highlighted as important stakeholders in vital statistics reporting in this context. Third, in the Vas, visible signs of emotional distress were assessed by interviewers, which may be subject to individual biases. Moreover, a clinician-assigned cause of death was not available. Finally, the small number of FGDs performed may represent only some of the perspectives of women and Imams in the community.

## Conclusion

Globally, there has been an increased focus on the importance of improving vital registration systems to enhance mortality reporting and facilitate the prioritisation of resources, particularly in regard to promoting child health. While child death was universally perceived to be painful, we found cultural norms that promoted religious acceptance of child loss and delegitimised other forms of speaking about or grieving such a loss. These norms must be taken into consideration when designing mortality surveillance systems that rely on a level of community reporting, including adapting VA tools to reflect locally acceptable terms used around death. Future policies could explore integrating Imams into local mortality-reporting strategies given their trusted role in these communities; however, special attention must be placed on ways to capture neonatal deaths and stillbirths in these reporting systems. In particular, understanding how early child deaths and stillbirths are perceived and mourned could improve how these deaths are counted.

## Paper Context

We explored perceptions of child deaths and socio-cultural factors influencing reporting of child deaths in Jigawa State, Nigeria, using a mixed-methods approach. We spoke to community members and Imams, who described culturally grounded perceptions of child death and multi-layered reasons for non-reporting. Religion plays a central role in traditional burial practices, mourning rituals, and how grief is expressed, but was not reported to have a role in reporting, providing an opportunity for vital registration.

## Appendix B. Focus Group Discussion Topic Guide, Round 1

FOCUS GROUP DISCUSSION GUIDE (ROUND 1)

**Kiyawa & Maje**

**Aim**: The aim of this interview is to access the culture and practices around the death of children using participatory methods. Participants are hereby urged to feel free to answer questions around this.

**Note to Interviewer**: Please use relevant questions as part of this process. If participants provide one-word answers, remember to probe around each for feelings, experiences and understanding. Should in case at any point participants feel emotional about any of the questions, please purse a bit and show empathy before you continue, and if the participant feels she cannot continue with the interview, you may stop.

1. How do people talk about death in this community?

Prompt for the following … … .

- Is it something people like to talk about? Why, why not?
- How do people describe it?
- What do people perceive it to be?

(2) Do people from this community have a special name for that aside from the usual or common name?
  a.If yes, what do people call it?
  b.Why do they call it so?
(3) Can you tell me things that are acceptable and not acceptable to say or do about death in this community? Please give reasons for that
(4) Do people like to be asked about the death of their loved ones? Why, why not
(5) How do people feel when asked about the death of their loved ones?
  a.Why do they feel this way?
(6) How do people feel about the death of a child?
  a.Do they see it as something painful? Why, why not
  b.Do they see it as something one should mourn? Why, why not
(7) Can you tell me two suitable ways to approach or ask someone about the death of a loved one without hurting the person?
(8) Are there things that are taboo about death? If yes, what are they?
(9) Can you tell me a bit about burial practices in this community?

Prompt for … … .

- Procedure to burial, how is it done?
- Who are permitted to attend? Why
- Do people see it as an avenue for celebration (i.e. eat, connect with friends, etc.)

(10) Do people really do the remembrance of loved ones? If yes, how is it done?

Prompt for … … …

- After how many years of departure is this done?
- What activities usually take place during remembrance?

Is there anything else you would like to say or talk about with me today?

## Appendix C. Focus Group Discussion Topic Guide, Round 2

FOCUS GROUP DISCUSSION GUIDE (ROUND 2)

**Kiyawa & Maje**

**Aim**: The aim of this interview is to gather information on better and acceptable ways to ask questions surrounding child’s death and to also prompt on ways through which death cases surrounding children could be reported. Participants should however bear in mind that we are not doing so to bring back old unpalatable or ugly memories, nor are we here to speak ill of the deceased, they should rather see it as a means to better inform health services and ensure future children are healthy and free from preventable childhood illnesses that could lead to death. On this note, we seek the indulgence of participants to feel free to answer questions regarding this.

**Note to Interviewer**: Please use relevant questions as part of this process. If participants provide one-word answers, remember to probe around each for feelings, experiences and understanding. Stories, scenarios, examples could also be shared to foster points.

1. Pregnancy Timeline Method

- What are the reasons that women would report or share details of the death of a child?
  - Would they report it when the event has to do with child death? Why/why not?
  - Would you report the death of an under-five child to your health facility, if it happens at home? Give reasons for your response
  - Would they report it if considered as something that one may not necessarily mourn? Why/why not?
  - On what other grounds would they report death events?
  - Would they be private/silent about the events? Why/why not?
  - What could be done to ensure death events are appropriately reported?
- Do you feel when asked questions around child’s death other household members like father could be present to help answer this or give emotional support? Give reasons for your response
- Could you sight questions that could help distinguish between stillbirths and live births?
- Could you sight better ways to ask these questions?

2. Prospective follow-up

As part of our objectives, we planned re-visiting compounds where compound and women questionnaires were administered about pregnancy, births and child survival. We have the intention of doing this every 4 months.

- Do you think this timing is okay? Give reasons for your response.
- If not, what time in months would be appropriate to do this? Give reasons for your response.
- How should information about under-five death be asked? That is, should it be done individually or with companions? Why?

- How early in pregnancy would women tell others?
- Is it something they keep private? If yes, why do they do that and how long do they tend to keep it private?
- Are there taboos we need to know about when asking a woman about their pregnancy? If yes, what are they?
- Is prayer or traditional medicine common for protection of early pregnancies? If yes, what are they?

3. Conduct

**Note to interviewer**: in quest to ensure the way we conduct our research is seen as trustworthy and women are free/comfortable to tell us about their children, you are required to explain who data collectors are and how they approach compounds. You may choose to define it this way if considered useful.

‘A data collector is a person usually an interviewer employed to get/collect information on pressing issues basically for research purpose. This is done to inform our decision on what is obtainable in a given setting. They carry out their duty by going from household to household seeking for consent from appropriate source before discharging their roles’.

The following questions are targeted towards how data collectors should appear, do or say especially when going into communities to ask questions relating to death events.

- What are the prayers said into the interview process when we learn of a death?
- Would this be appropriate for us to do? Give reasons for your response
- What sort of condolence do you feel data collectors should give, especially if they are not well known to the family?
- How important is data collectors’ attire when they go for condolence visit interviews?
- How do you think they should be dressed (i.e. what should they appear in)?
- Do you think it is appropriate to come in make-up and perfumes? Why/why not?

**Closing question**: is there anything we have not mentioned about death events (particularly child’s death events) that you would like to discuss with us?

## Appendix D. Key Informant Interview Topic Guide

KEY INFORMANT INTERVIEW TOPIC GUIDE

**Kiyawa & Maje**

**Introduction**: Peace be unto you. We are **(interviewers should introduce themselves and where they are from**). We invited you here today for a round of interviews because we see you as an important stakeholder in the community that should be acknowledged. The purpose of this interview is to have a better understanding of your roles (as an Imam) on community health, most importantly child’s health and to also know the culture and practices around child’s death. Your contributions/opinion on the subject of discussion are highly respected.

**Note to Interviewer**: Please use relevant questions as part of this process. If participants provide one-word answers, remember to probe around each for feelings, experiences and understanding. Should at any points participants feel some questions are not meant to be answered probably due to religious reasons, please respect their feelings.

1. Where were you born?
  - How long have you been here?
2. How did you decide to become an imam?
  - Have you been an imam in any other community?
    - If yes, was it different from being an imam here?
    - What makes the difference (Explain)?
3. Can you tell us about the mosque in the community?
  - What does it look like?
  - How many people does it serve?
  - How old is it?
  - What role does the mosque play in the community?
4. What is a typical day like for you?
  - Does this change for holy days? (Fridays, Eid)
    - If yes, how is it different?
5. What is the biggest challenge of being an imam in this community?
6. How do you see your role in the community?
  - What type of activities do you do in the community?
  - What kinds of events are you involved in?
    - Do you help when community projects come here?
    - What role do you play in community health?
    - Do you provide information about your health?
    - If yes, what information have you provided/passed about health? (We could also rearrange the order of the questions, i.e. 1,2,3,4,6,7,5 & 8)
    - How did you go about that?
7. What happens when a person dies in the community?
  - What is your role at a funeral?
  - Is there a different process when a child dies?
  - Does your role change if a child has died?
  - Do you record deaths?
    - If yes, why do you do that?
    - Is there a register in the mosque for that?
    - If no, why not? (Probe for cultural/religious reasons)
8. Overall, what is the best aspect about being an imam in this community?

**Closing question**: is there anything you would like to say or talk about with us today?
